# Life Cycle Assessments in Healthcare: Insights and Standardisation Needs

**DOI:** 10.3390/ijerph23070828

**Published:** 2026-06-23

**Authors:** Franziska Zecha, Lena-Marie Hupperich, Tobias Viere

**Affiliations:** 1 Institute for Industrial Ecology, Pforzheim University, Tiefenbronner Str. 65, 75175 Pforzheim, Germany; 2 School of social sciences, Saarland University of Applied Sciences, Goebenstr. 40, 66117 Saarbruecken, Germany; lena-marie.hupperich@htwsaar.de

**Keywords:** life cycle assessment, healthcare sustainability, health system decarbonisation, standardisation, carbon footprint, healthcare services, product category rules, environmental health

## Abstract

**Highlights:**

**Public health relevance—How does this work relate to a public health issue?**

- Healthcare delivery contributes substantially to greenhouse gas emissions, linking healthcare operations to population health risks from climate change.
- Life cycle assessment is increasingly used to quantify these impacts, but inconsistent application limits its usefulness for health system decision-making.

**Public health significance—Why is this work of significance to public health?**

- Methodological heterogeneity prevents aggregation and comparison of healthcare LCA results, constraining evidence-based decarbonisation of health systems.
- This study identifies where standardisation is currently most feasible and where healthcare-specific guidance is urgently needed to support public health policy.

**Public health implications—What are the key implications or messages for practitioners, policy makers and/or researchers in public health?**

- Product-level LCA already provides comparatively consistent evidence and can support sustainable healthcare procurement.
- To inform clinical and system-level decisions, service and organisational assessments require pathway-based functional units and defined hospital sub-unit coverage.

**Abstract:**

Life cycle assessment is increasingly applied in healthcare, yet the healthcare-specific standardisation landscape and its relation to current practice remain unclear. This study maps existing frameworks and analyses their alignment with published healthcare LCA to identify standardisation gaps. Healthcare-specific standards and product category rules were identified through grey literature searches. Published healthcare LCA studies were quantitatively analysed and compared with the identified frameworks to assess methodological convergence and divergence. Six healthcare-specific frameworks were identified: five address medical products, one addresses services, and none cover organisational assessment. Product-level applications showed strong alignment in structural modelling elements including system boundaries and life cycle stages, while substantial heterogeneity persisted in functional unit definitions and impact assessment approaches. Service and organisational assessments showed broader variability in modelling approaches, functional units, and system boundary conceptualisations, indicating distinct modelling logics of healthcare delivery across assessment levels. Healthcare LCA practice is consistent with ISO-based principles but lacks a shared conceptual modelling logic for healthcare delivery systems. Rather than reflecting a single methodological paradigm, healthcare LCA combines product-, intervention-, pathway-, and organisational-oriented approaches. Standardisation efforts should therefore focus not only on harmonising calculation methods but also on developing healthcare-specific modelling conventions for products, services, and organisational structures.

## 1. Introduction

The healthcare sector is a major contributor to global greenhouse gas emissions, accounting for around 4–5% of global totals and up to 7% of national emissions in several European countries [1,2,3,4]. A substantial share arises from the production, use, and disposal of medical devices. Life cycle assessment (LCA) provides a systematic framework for evaluating environmental impacts across the entire life cycle and is recognised as a tool for identifying emission hotspots, optimising supply chains, and informing sustainable procurement [5,6].

The relevance of LCA extends beyond scientific research, as it now underpins policy measures and tools (e.g., EU Taxonomy, Digital Product Passports, Environmental Product Declarations) [7,8]. LCA operates at product and organisational levels to quantify environmental impacts from raw material extraction to end-of-life scenarios. Initially, LCA focused on products, aiming to capture all relevant environmental impacts (e.g., climate change, eutrophication, acidification, eco-toxicity). LCA has been extended to organisational assessments and corporate carbon footprints, and to evaluations that focus on specific environmental impacts only, such as water footprints or product carbon footprints [9,10]. Furthermore, LCA is combined with social and economic considerations, enabling a Life Cycle Sustainability Assessment [11]. Product category rules are being developed to enable environmental product declarations and product Environmental Footprints, to enhance comparability of LCA results within the same product group [12,13].

LCA results inform political, managerial, and clinical decisions in healthcare. LCA has been used to assess environmental trade-offs between single-use and reusable medical devices, providing recommendations for hospitals on whether to switch to alternatives [14,15]. LCA is applied to guide procurement decisions for medical products and to enable environmental and greenhouse gas reporting within hospitals [16,17,18]. On the producer side, LCA supports circular and eco-design of medical products by identifying hotspots [19,20].

Alongside product- and organisational-level assessments, service LCA play a central role. Healthcare systems are service-oriented, with clinical treatments and care pathways constituting a third, distinct LCA category. While product LCA is supported by standards such as ISO-14040, ISO-14044, ISO-14067, ISO-14025 and PAS-2050 and the organisational level is addressed through frameworks such as ISO-14072 and ISO-14069, no methodological framework exists for LCA of healthcare services [5,6,10,21,22,23].

At the same time, the importance of LCA in healthcare is growing, and new standardisation initiatives are emerging. The German Institute for Standardisation has established a working group on LCA of medical devices [24], and the British Standards Institution has released a draft standard for applying LCA to pharmaceutical products [25]. These developments underline the need to systematically identify applicable standards and examine how they are applied in practice to guide methodological harmonisation and improve comparability across studies.

A recent Technical Brief by the US Agency for Healthcare Research and Quality (AHRQ Technical Brief) [26] shows that a substantial number of LCA have already been conducted across different healthcare contexts. The brief provides a descriptive overview of LCA applications, based on a literature search and expert input. It offers a broad overview of current practice but does not explicitly assess the use of formal LCA standards or frameworks in healthcare, nor does it systematically distinguish between different types of LCA or discuss key methodological aspects (e.g., functional units, cut-off criteria, data assumptions). The methodological architecture of healthcare LCA remains poorly characterised. Although several standardisation initiatives and methodological guidance documents have emerged for specific healthcare-related LCA applications, it remains unclear to what extent these approaches are reflected in published healthcare LCA practice. As a result, comparability and reproducibility between studies may remain limited, although both are essential prerequisites for translating healthcare LCA results into procurement, policy, and regulatory decision-making [27].

In particular, the conceptual distinction and operationalisation of different forms of healthcare LCA have not yet been systematically examined. Without methodological harmonisation, LCA results risk becoming difficult to compare, interpret, and integrate into procurement, policy, and regulatory decision-making.

The objective of this paper is to empirically examine how LCA is currently implemented and operationalised in the healthcare sector based on published LCA studies and to assess the extent to which standardised or harmonised methodological approaches are applied. By systematically analysing existing LCA studies in healthcare, this work aims to identify prevailing methodological choices, evaluate their alignment with established standards and frameworks, and reveal areas where methodological gaps or further standardisation needs become apparent.

To achieve this objective, the study addresses the following research questions:

1. Which sector-specific standards and frameworks for LCA have been developed for application in healthcare?
2. How is LCA currently conceptualised and operationalised across different assessment levels and healthcare contexts?
3. To what extent does current practice align with established standards and frameworks, and where do methodological inconsistencies or needs for further standardisation become apparent?

## 2. Methods

Sector-specific standards and healthcare-oriented product category rules (PCRs) were identified through a structured grey literature search. Sources included standardisation bodies and international standard initiatives, environmental declaration programme operators and PCR registries, as well as backward reference screening and targeted searches of organisational websites on recognised institutions. The search was conducted between April and November 2025 using internet search engines in English. Search terms included combinations of “life cycle assessment”, “healthcare”, “medical devices”, “pharmaceuticals”, “carbon footprint”, “product category rules”, “environmental footprint”, “guideline” and “standard”. Documents were included if they provided methodological guidance for conducting LCA or carbon footprinting of healthcare products, services, or organisations. PCRs act as subordinate LCA standards that specify methodological rules for performing LCA and issuing environmental declarations for product groups.

**Figure 1:**
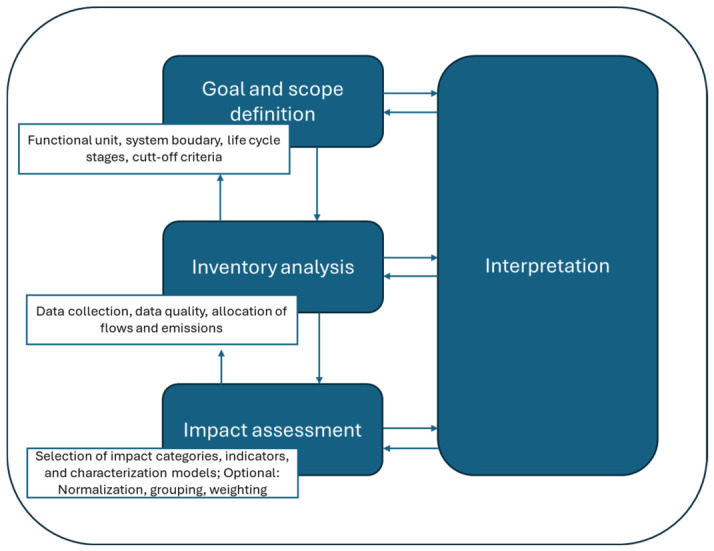
General LCA structure (adapted from ISO-14040/44). Alt text: Schematic adaptation of the ISO-14040/44 framework illustrating the four main phases: goal and scope definition, inventory analysis, impact assessment, and interpretation. Each phase highlights key methodological elements. Bidirectional arrows illustrate the iterative nature of the process, with interpretation interacting with all phases.

General cross-sector LCA and carbon footprint standards were screened but not included in the qualitative framework analysis, as they do not provide healthcare-specific methodological guidance. Nevertheless, their underlying LCA principles informed the analytical reference structure (Figure 1), which was used to assess and compare the methodological characteristics of healthcare-specific standards.

In parallel, a structured identification of LCA studies was conducted. Scientific studies were obtained from the HealthcareLCA Database and complemented by reference screening of literature-based meta-studies to verify completeness. After removal of duplicates, records were screened for relevance. Studies were included if they assessed healthcare delivery, medical products, healthcare services, or healthcare organisational processes using life cycle assessment methodology. Studies evaluating research-related activities (e.g., clinical trials, conferences, or educational training events) were excluded because they represent knowledge generation rather than healthcare delivery and therefore fall outside the defined system boundaries. Additional exclusions comprised LCA of packaging and food products not directly related to medical care.

**Figure 2:**
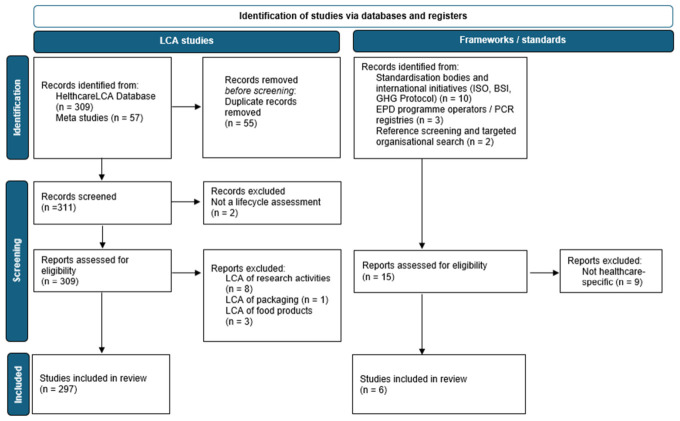
Adapted PRISMA 2020 flow diagram of the identification and selection process for LCA studies and healthcare-specific frameworks (adapted) [28]. Alt text: Flow diagram showing the identification and selection of healthcare life cycle assessment (LCA) studies and healthcare-specific methodological frameworks. For LCA studies, 309 records from the HealthcareLCA Database and 57 from meta-studies were screened; after duplicate removal and eligibility assessment, 297 studies were included. Exclusions comprised non-LCA studies and LCA of research activities, packaging, and food products. For standards and frameworks, 15 documents identified through standardisation bodies, PCR registries, and reference searches were assessed, of which 6 healthcare-specific frameworks were included after excluding non-healthcare-specific documents.

The selection process for both LCA studies and standards is documented in Figure 2.

A quantitative content analysis of LCA studies was conducted using univariate descriptive statistics (frequency distributions and percentages). Each study was classified as organisational, service, or product LCA (see Section 3.2). In line with the framework analysis, each LCA study was examined using the general LCA structure (Figure 1). Where applicable, additional characteristics such as medical speciality and background databases were recorded.

This study combines two complementary sources of evidence: empirical evidence from healthcare LCA studies and normative evidence from sector-specific standards and product category rules. This dual perspective allows assessment of both current LCA practice and its methodological framework, enabling identification of standardisation gaps in healthcare LCA practice.

## 3. Results

### 3.1. Healthcare-Specific LCA Standards

Six healthcare-specific standards and guidelines were identified.

- The second edition of *Care Pathways: Guidance on Appraising Sustainability* (*Care-Pathways*) provides service-level guidance for healthcare pathways [29].
- *Greenhouse Gas Accounting Sector Guidance for Pharmaceutical Products and Medical Devices* (*GHG-P&M*) offers product-level guidance aligned with the GHG Protocol Product Life Cycle Standard [30]
- *Product Category Rules 2016:07 Blood and blood-derived products for therapeutic or prophylactic uses* (*PCR-Blood*) define rules for blood-related products [31].
- *Product Category Rules 2017:01 Disposable Surgical Drapes, Gowns, Air Suits and Face Masks* (*PCR-PPE*) define LCA rules for personal protective equipment [32].
- *Product Category Rules for pharmaceutical products and processes (PCR-Pharma)* is considered an academic, whitepaper-style framework rather than a fully operational PCR [33].
- *Publicly Available Specification 2090 Pharmaceutical products—Product category rules for environmental life cycle assessments of pharmaceuticals* (*PAS-2090*) is a recently published British Standard [25].

Five of the six healthcare-specific standards relate to products, three of which are limited to pharmaceuticals (*PAS-2090*, *PCR-Pharma*, *PCR-Blood*), while *PCR-PPE* is limited to personal protective equipment. Only *GHG-P&M* provides cross-category coverage for medical devices and pharmaceuticals. For services, the *Care-Pathways* framework is the only available healthcare-specific guidance, and there is no sector-specific guidance for organisational LCA. A detailed comparison of all standards along the LCA structure from Figure 1 is provided in Supplementary Material and summarised below.

All standards define the use of functional units. *PCR-Blood and PCR-PPE* use one clearly defined functional unit each (single dose of product and one product unit, respectively). *GHG-P&M* allows the functional unit to be defined per product or per use. *PAS-2090* allows both a functional unit based on product quantity or treatment. *Care-Pathways* and *PCR-Pharma* adopt an integrated approach derived from a set of clinical and contextual parameters that are included in the functional unit for therapeutic treatment.

All standards require examination of the entire life cycle (cradle-to-grave), although *GHG-P&M*, *Care-Pathways* and *PAS-2090* also allow cradle-to-gate studies. Most standards use a generic classification of life cycle stages (e.g., raw material extraction, production, distribution). *Care-Pathways* instead follows a modular patient pathway approach (e.g., patient travel, emergency department visit) to model services.

Cut-off criteria for permissible exclusion of material flows range from 1% (*GHG-P&M*, *PCR-PPE*, *PCR-Pharma*, *PCR-Blood*) to 2% (*PAS-2090*). The sum of all excluded flows should not exceed 5% in *GHG-P&M* and *PCR-Pharma*, whereas the 1% limitation in *PCR-PPE* serves as the limit. The *Care-Pathways* does not define a threshold but cites 1% and 10% as examples.

Data quality requirements differ. *GHG-P&M*, *all PCR*, *and PAS-2090* mandate quantitative or semi-quantitative assessment, often using pedigree matrices for structured evaluation of data reliability. *Care-Pathways* relies on qualitative assessment only. *PCR-Pharma* defines specific temporal requirements with primary data expected to be no older than three years and secondary data no older than five years.

All frameworks distinguish between primary data, i.e., self-researched, accurate process data, and secondary data, i.e., literature- and database-based generic data. *PCR-PPE*, *PAS-2090*, *PCR-Pharma*, and *PCR-Blood* provide detailed guidance on which processes require specific data, including scenario modelling for transport, packaging, electricity mix, and end-of-life stages.

All examined frameworks follow general LCA allocation procedures and prefer allocation based on physical properties over economic allocation.

Impact assessment coverage also diverges. *GHG-P&M* restricts itself to carbon footprinting, while other frameworks, especially *PAS-2090,* prescribe a broader set of indicators. The treatment of optional impact assessment elements such as normalisation, grouping, and weighting varies. *Care-Pathways* and *GHG-P&M* provide no specific guidance. *PCR-PPE* and *PCR-Blood* prohibit their use in environmental product declaration. *PAS-2090* recommends the PEF method, while *PCR-Pharma* allows these practices only for internal use.

### 3.2. LCA Studies

The case study review comprised 309 studies derived from the HealthcareLCA Database [34]. Comparison with published healthcare-related LCA meta-analyses product [35], service [36] and organisational [37] LCA showed a very high overlap, with only two studies not represented in the dataset. These studies did not report original LCA but relied on previously published results [38,39] and were therefore irrelevant for analysing applied assessment parameters. Overall, the comparison indicates a high level of completeness of the database for the present analysis.

**Figure 3:**
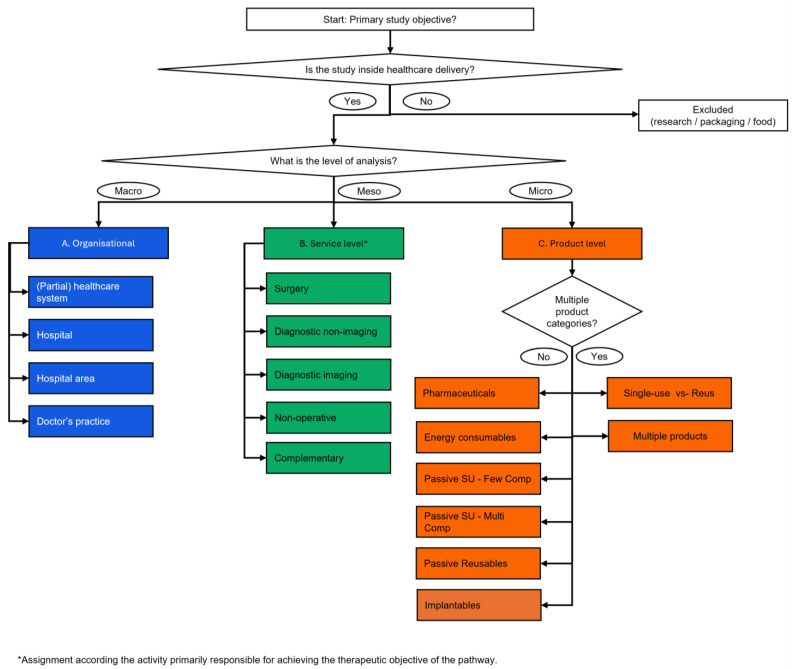
Decision tree for clustering the studies into the three main categories and subcategories. Alt text: Flowchart of the classification scheme for healthcare LCA studies. Studies are first screened for inclusion within healthcare delivery; others (e.g., research, packaging-only, or food-related) are excluded. Included studies are classified by level of analysis into organisational (macro), service (meso), or product (micro). Service categories are assigned based on the dominant clinical activity (e.g., surgery, diagnostics, non-operative, complementary), while product categories follow functional device and pharmaceutical groupings, including single-use, reusable, and implantable products.

After applying the predefined eligibility criteria (Section 2), 297 studies remained for classification and further analysis (Figure 2). All LCA studies were classified into organisational, service and product LCA according to the decision tree in Figure 3.

**Figure 4:**
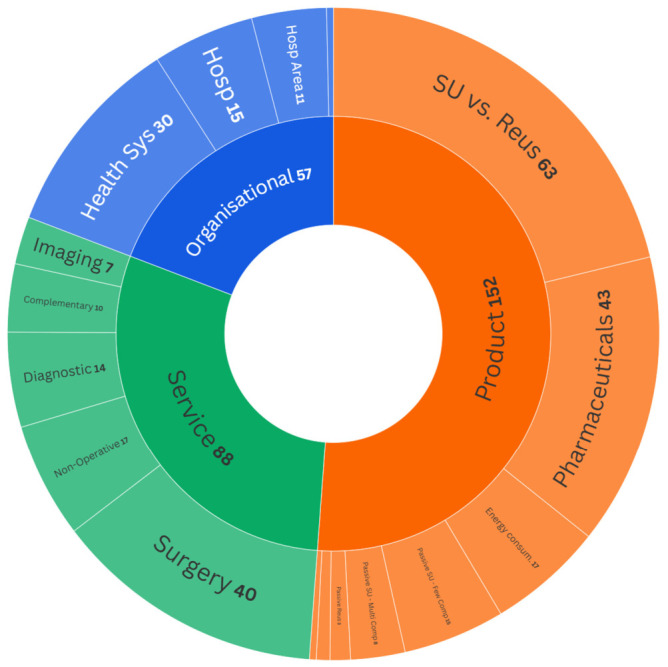
Distribution of healthcare LCA studies. Alt text: Circular classification diagram of 297 healthcare LCA studies grouped into three categories: product LCA (152 studies), service LCA (88 studies), and organisational LCA (57 studies). Product LCAs are mainly pharmaceuticals and single-use versus reusable products. Service LCAs are dominated by surgical procedures, followed by non-operative and diagnostic services. Organisational LCAs include health systems and hospital-level assessments.

Based on the classification framework shown in Figure 3, studies were grouped into organisational-, service-, and product-level assessments, with the resulting distribution visualised in Figure 4.

The organisational *category* (*n* = 57) includes LCA of healthcare units, such as health systems, hospitals, hospital functional areas, or medical practices.

The service category (*n* = 88) was further grouped by medical and supportive services using the German Procedure Classification (Operationen- und Prozedurenschlüssel OPS), which adapts the World Health Organisation’s former International Classification of Procedures in Medicine, which is intended to be replaced by the International Classification of Health Interventions, currently under development [40].

For products (*n* = 152), subcategories largely correspond to *GHG-P&M* [25], namely: passive, single-use devices with multiple components/materials; passive, single-use devices with few components/materials; passive, reusable devices; implantable devices; energy-consuming devices; and pharmaceuticals. Some studies were not assigned to a single subcategory because they covered multiple groups or compared disposable versus reusable devices (cp. Figure 3).

**Figure 5:**
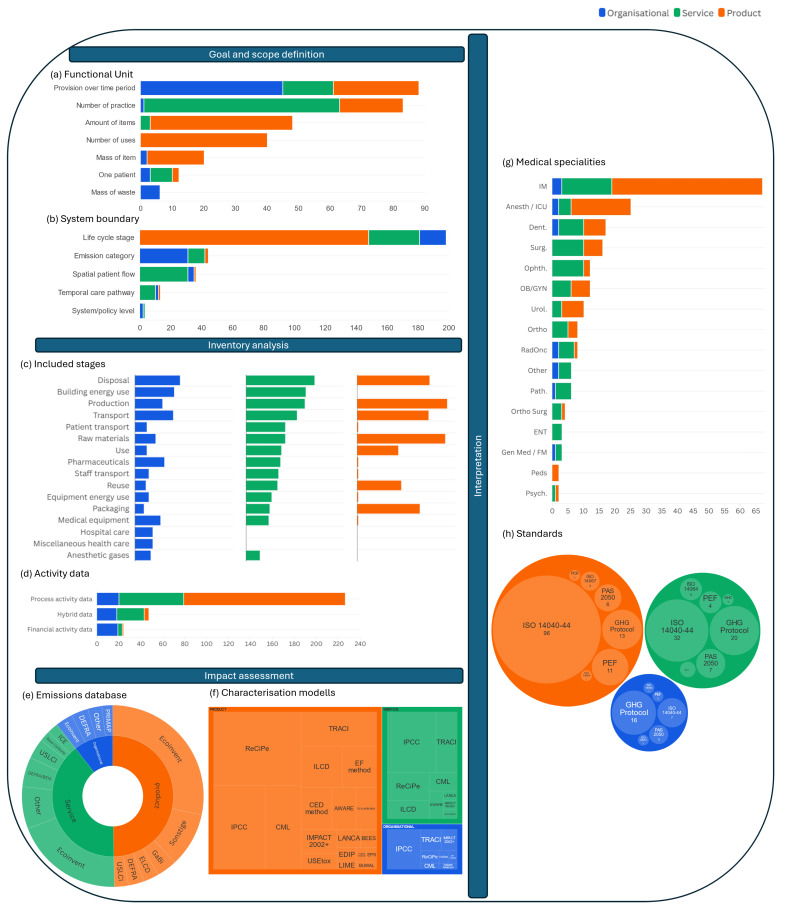
Evaluation of the studies organised according to LCA phases with (**a**) distribution of functional units, (**b**) determination of system boundaries, (**c**) included stages, (**d**) sourcing of data types, (**e**) employment of background databases, (**f**) distribution of characterisation modells, (**g**) frequency of medical disciplines, (**h**) application of standards. Alt text: bar charts and grouped plots showing methodological choices in healthcare LCA studies, organised by type of LCA (organisational, service, product).

Figure 5 analyses the LCA studies across the three principal categories, considering differences among subcategories and additional aspects, including medical discipline, background database, and software.

Functional units were classified according to the primary functional reference explicitly emphasised in the study design. Classification followed a hierarchical decision logic distinguishing between provision-oriented, patient-oriented, practice-oriented, use-oriented, item-oriented, and mass-oriented functional units. In cases where multiple dimensions were present (e.g., a product used during a procedure over time), classification was assigned according to the dominant functional framing of the functional unit. Detailed clustering rules and examples are provided in the Supplementary Materials.

Organisational studies were predominantly defined by provision-oriented functional units over a time period, whereas service studies were primarily characterised by practice-based functional units (Figure 5a). Product studies showed the greatest heterogeneity and were mainly characterised by amount-based functional units and use-based functional units, followed by provision-oriented, practice-based, and mass-based units.

Classification followed a hierarchical decision logic distinguishing between: (1) temporal care pathways, defined by the chronological progression of patient care over time; (2) spatial patient flows, defined by physically or organisationally delimited healthcare settings, locations, or transitions; (3) clinical processes, defined by discrete clinical procedures, treatment activities, or healthcare processes; (4) life cycle stages, defined by product or service life cycle phases (e.g., cradle-to-gate, cradle-to-grave); (5) emission categories, defined by greenhouse gas accounting scopes; and (6) system-/policy-level boundaries, defined at the level of healthcare systems, policy frameworks, or sector-wide service provision. In cases where multiple dimensions were present, classification was assigned according to the dominant boundary framing described in the study. Detailed classification rules and examples are provided in the Supplementary Materials.

Organisational studies were predominantly defined using emission-category boundaries, followed by life cycle stage approaches. Service studies showed the greatest heterogeneity, with life cycle stage boundaries and spatial patient flow boundaries occurring most frequently, followed by emission-category approaches and temporal care pathway boundaries. Product studies were overwhelmingly characterised by life cycle stage boundaries, primarily reflecting cradle-to-gate, cradle-to-use, and cradle-to-grave approaches (Figure 5b).

LCA studies often group processes and activities into life cycle stages, such as raw material production, manufacturing, distribution, use and end-of-life. Figure 5c depicts the prevalent stages in healthcare LCA studies. Product and organisational studies exhibit a rather focused selection of stages, whereas service studies are more heterogeneous. Inventory data collection and calculation methods (Figure 5d) are dominated by process-based physical data. Service LCA primarily use process data, but often employs hybrid approaches combining physical process and financial data. Organisational studies use process, hybrid, and financial data in roughly equal measure. Regarding software usage, SimaPro is frequently used across all study types, followed by OpenLCA. Gabi and Umberto are almost exclusively used in product studies, whereas Bilan Carbone and Ansys Granta EduPack are primarily used for service studies.

For background data (Figure 5e), product and service studies mainly rely on the life cycle inventory database ecoinvent, whereas organisational studies show no clear trend. Within the impact assessment phase (Figure 5f), product studies employ a range of characterisation methods, most notably ReCiPe, TRACI, IPCC and CML, alongside ILCD, EF method, CED method, AWARE, Eco-indicator, IMPACT 2002+, USEtox and other methods. Service and organisational studies focus more on IPCC, TRACI, ReCiPe, and IMPACT 2002+.

In the interpretation phase, comparative and contribution analyses are most frequent in product and organisational studies. Product studies commonly apply multiple analyses, including sensitivity, scenario, and uncertainty analyses, whereas service studies predominantly use comparative and sensitivity analyses.

Internal medicine is the most represented medical field across all categories (Figure 5g). Product studies are also frequently conducted in intensive care, while service studies are common in surgery and ophthalmology. Organisational studies show no distinct disciplinary focus.

ISO-14040/44 is the most frequently used standard in product studies, while the GHG Protocol dominates in organisational studies (Figure 5h). Service studies refer to both standards, with no clear dominance.

### 3.3. Alignment of Healthcare LCA Practice with Existing Standards

To identify needs for further alignment and standardisation, current LCA practice as described above was compared with healthcare-specific standards from Section 3.1.

To date, no sector-specific standards exist for organisational LCA in healthcare. Consequently, studies in this area rely on generic frameworks such as the GHG Protocol for corporate carbon footprints and its classification of emissions into scopes [22]. For services, *Care-Pathways* provides a methodological framework, which is compared to actual LCA implementation in Table 1.

Overall, the comparison indicates partial rather than complete alignment between existing guidance and current practice in healthcare service LCA. While both approaches generally apply cradle-to-grave thinking and include multiple life cycle stages, practice operationalises functional units and system boundaries more heterogeneously and frequently combines life cycle-based with spatial or process-oriented boundary logics. In particular, service-based studies often rely on procedure- or activity-oriented functional units rather than modular patient pathway definitions proposed in conceptual guidance.

In contrast to service LCA, product LCA show a substantially higher alignment between standards and practice. This likely reflects the presence of multiple product-oriented frameworks (e.g., *PCR-Blood*, *PCR-Pharma*, *PCR-PPE*) that provide more operationalised methodological guidance for specific applications. Comparable differentiated guidance is largely absent for healthcare services and organisational LCA. However, as existing product category rules cover only a subset of healthcare products, *GHG-P&M* remains the only broadly applicable healthcare-specific reference framework and is therefore used as the main reference in Table 2.

Overall, product-level medical LCA showed alignment with GHG-P&M for several methodological elements. Alignment was particularly strong for system boundaries, life cycle stages, and inventory modelling approaches. However, substantial heterogeneity was observed in the operationalisation of functional units, which ranged from item-, use-, and practice-based definitions to mass-based pharmaceutical functional units. Additional variation was observed in the selection of impact assessment categories, as practice frequently extended beyond the limitation of the carbon-footprint logic in GHG-P&M. Compared with healthcare service LCA, alignment between framework and practice appeared stronger for certain structural LCA elements, whereas considerable variation remained in the conceptualisation of functional units and impact assessment approaches.

## 4. Discussion and Limitations

This research provides a systematic comparison of healthcare LCA practice at product, service, and organisational levels against existing sector-specific LCA standards.

Both this analysis and the AHRQ Technical Brief indicate that, despite a growing body of healthcare LCA, methodological heterogeneity remains a characteristic [26]. While the AHRQ Technical Brief emphasises the need for standardised reporting and improved comparability, it does not systematically differentiate between levels of assessment. By explicitly distinguishing product-, service-, and organisational-level practice, this analysis shows that rather than following a single modelling logic, healthcare LCA currently combines different conceptualisations of healthcare within LCA modelling, including product-oriented, intervention-oriented, pathway-oriented, and organisational accounting approaches.

This pattern was also reflected in the distribution of functional unit and system boundary types across assessment levels. Product studies predominantly applied item-, use-, and life cycle stage-oriented modelling approaches, closely resembling conventional product-system logic in LCA. In contrast, service studies combine intervention-oriented functional units (e.g., procedures, treatments, appointments) with both pathway-/spatially oriented and conventional life cycle stage boundaries, indicating a hybrid modelling structure. Organisational studies were primarily characterised by provision-oriented functional units over time and emission-category boundaries, reflecting institutional and capacity-oriented accounting perspectives rather than discrete product systems.

Interestingly, functional unit definitions appeared conceptually more homogeneous at service and organisational level than at product level. While product studies applied highly diverse item-, use-, mass-, practice- and provision-based functional references, service studies predominantly relied on practice-oriented units (e.g., procedures, appointments, treatments), and organisational studies largely used provision-oriented units over defined time periods. This suggests that methodological heterogeneity in healthcare LCA is unevenly distributed across methodological dimensions. While product studies showed relatively consistent structural modelling approaches, they simultaneously exhibited highly heterogeneous functional unit definitions.

A strength of GHG-P&M lies in its explicit differentiation between medical devices and pharmaceuticals through multiple product categories, reflecting differences in production processes and supporting improved accuracy and comparability. Since GHG-P&M is designed as a carbon footprint standard, its scope is limited to greenhouse gas emissions and does not address broader environmental impacts. In addition, functional units at the product level remained highly heterogeneous and were operationalised inconsistently across studies. Overall, tangible medical products correspond relatively well to established product system modelling, whereas healthcare services and organisations represent complex socio-technical systems composed of interacting processes and actors. Consequently, many inconsistencies observed in healthcare LCA do not arise from analytical calculation methods but from the absence of a shared conceptual model of healthcare delivery within LCA practice.

Service-level healthcare LCA were characterised by substantial conceptual and methodological heterogeneity. The *Care-Pathways* framework offers an ISO-consistent, modular logic that defines functional units via patient, disease, location, and time characteristics and structures systems into pathway modules (e.g., consultations, inpatient days, procedures). In practice, service studies frequently combined different functional reference logics and system boundary framings. Functional units are predominantly intervention-oriented (e.g., “one procedure”, “one treatment”, or “one appointment”), while system boundaries combine both healthcare-specific pathway/spatial logic and conventional life cycle stage approaches such as cradle-to-grave modelling. This hybrid structure indicates that service-level healthcare LCA currently operates between classical product-system modelling and pathway-oriented representations of healthcare delivery.

The divergence between guidance and practice indicates a deeper structural issue: healthcare services are not single processes but coordinated care pathways involving multiple actors, locations, and temporal stages. Conventional LCA modelling approaches, which typically analyse discrete product systems, therefore struggle to represent healthcare delivery adequately. In line with the AHRQ Technical Brief, the findings suggest that many healthcare LCAs remain research-driven and are not yet embedded as routine tools in clinical or organisational decision-making [26].

Despite the limited practical application, the *Care-Pathways* framework demonstrates potential. Service LCA could be improved by adopting consistent functional unit definitions, clearly defined system boundaries, and structured pathway modules. Similar to product categorisation in *GHG-P&M*, healthcare services could be divided into subcategories to identify methodological commonalities and refine assessment approaches.

Organisational LCA in healthcare exhibits high methodological heterogeneity. Unlike product LCA, which is supported by detailed sector-specific guidance, or service LCA, which at least has a modular framework, no healthcare-specific standards exist for organisations, and studies rely primarily on general frameworks such as *ISO-14072* or the *GHG Protocol* (cp. Section 3.1 and Section 3.3). This reliance on cross-sector standards introduces variability in system boundaries, data collection approaches (process, hybrid, or financial), and impact assessment coverage (Section 3.2). Healthcare organisations do not operate as single production systems with a defined product flow but as coordinated care pathways combining multiple functional units. Organisational LCA approaches developed for product-based industries may therefore insufficiently represent environmental impacts in healthcare settings. This difference was also reflected in the predominance of provision-oriented functional units over extended time periods (e.g., one year of service provision), indicating that organisational studies primarily conceptualise healthcare as continuous system capacity rather than as discrete products or clinical interventions. This indicates that organisational healthcare LCA conceptually resemble institutional accounting systems more closely than conventional product-system LCA.

A notable distinction from corporate contexts is that analyses frequently extend to organisational sub-units rather than being confined to the organisation as a whole. Unlike conventional companies, healthcare organisations require accounting at ward or functional area level. This indicates that relevant environmental impacts cannot be adequately captured at the hospital level alone and highlights a demand for sub-unit-based accounting.

This study and the AHRQ Technical Brief converge on the conclusion that sector-wide progress in healthcare decarbonisation will require standardised, healthcare-specific LCA guidance. While product-level standards provide a useful reference point, service- and organisational-level assessments demand frameworks that are both operationally applicable and sensitive to the structural and contextual complexity of healthcare systems. Sector-wide progress in healthcare decarbonisation will therefore depend not only on harmonising calculation methods but also on developing shared modelling conventions for representing healthcare delivery, including pathway-based functional units, structured service modules, and sub-unit organisational accounting. Future guidance could extend beyond carbon footprint assessment and build on experience from other industries, where sector-specific methodological consensus is developed. At the same time, international collaboration is increasingly recognised as a key enabler for healthcare decarbonisation. Global analyses demonstrate the substantial contribution of healthcare systems to greenhouse gas emissions and associated health burdens [1,2,4], while initiatives at OECD and European level emphasise the need for harmonised methodologies and cross-country comparability [7,10,15]. Aligning healthcare LCA practice with established international standards (e.g., ISO 14040/44) and emerging frameworks such as the Environmental Footprint approach may therefore support more consistent and scalable application across healthcare systems [8,9,15].

The applied classification frameworks for functional units and system boundaries necessarily reduced multidimensional study characteristics to dominant conceptual categories. In practice, many studies simultaneously contained multiple functional and boundary dimensions (e.g., product use during procedures over time or pathway analyses combined with cradle-to-grave boundaries). Although hierarchical decision rules were applied consistently, alternative classifications may have been possible in some borderline cases. This ambiguity was itself an important finding, demonstrating that healthcare LCA frequently operate across overlapping conceptual reference dimensions rather than within clearly separated methodological categories. The resulting categories should therefore be understood as analytical heuristics intended to support comparison across studies rather than as fixed ontological distinctions. To ensure transparency, the complete classification dataset, including all study-specific assignments and clustering rules, is provided in the Supplementary Materials. This study is confined to the peer-reviewed scientific literature. Grey literature such as corporate carbon footprints and internal industry assessments was excluded, so the findings primarily represent academically documented practice. Only studies that reported sufficient detail could be coded and compared. Heterogeneous and sometimes incomplete reporting may have led to underrepresentation of certain methodological variants and introduces uncertainty into specific classifications (e.g., data types or boundary definitions).

## 5. Conclusions

Healthcare-related LCA is gaining importance, while exhibiting substantial methodological heterogeneity in its application. Substantial methodological differences exist between product-, service-, and organisational-level applications. This suggests that the central challenge of healthcare LCA is not primarily analytical methodology but the absence of a shared representation of healthcare delivery within LCA modelling. Rather than reflecting a single methodological paradigm, healthcare LCA currently combines product-system, intervention-oriented, pathway-oriented, and institutional accounting perspectives.

Consequently, standardisation efforts should focus not only on harmonising calculation procedures but also on establishing modelling conventions for healthcare pathways and organisational care structures. Product LCA showed comparatively strong alignment with *GHG-P&M* regarding structural modelling approaches but requires functional unit alignment and systematic extension beyond greenhouse gas emissions to support comprehensive, multi-impact assessment aligned with broader environmental policy and procurement needs. Service LCA frequently combined conventional product-system modelling with pathway-oriented representations of healthcare delivery, resulting in hybrid modelling structures and heterogeneous logic in functional unit and boundary definitions despite the availability of the *Care-Pathways* framework. Translating its conceptual, modular pathway logic into operationalised functional units, mandatory definition of pathway modules, such as OPS-based clustering, and explicit criteria for boundary and model selection, represents a key opportunity for harmonisation. Organisational LCA, in turn, requires foundational healthcare-specific guidance that clarifies relevant units of analysis, defines minimum process coverage, and specifies how Scope 1–3 concepts should be adapted to hospitals and sub-organisational units.

Across all three levels, improving comparability will require not only harmonisation of methodological procedures but also greater conceptual consistency in how healthcare activities, pathways, products, and organisational services are represented within LCA models. More fundamentally, the findings indicate that healthcare LCA currently lack a common epistemic framing of what constitutes the analysed system itself. Depending on the level of assessment, healthcare is implicitly conceptualised as a product, a clinical intervention, a patient pathway, or an institutional service system. These different representations are associated with distinct functional units, system boundaries, and modelling assumptions and therefore lead to fundamentally different forms of LCA implementation. Establishing greater conceptual consistency is essential to enable robust and comparable sustainability assessments in the healthcare sector and to support procurement, clinical and managerial decision-making, and alignment with emerging regulatory frameworks. Future work should integrate practice-oriented documents, empirically test modular service and organisational LCA concepts, and co-develop healthcare-specific rules for product, service, and organisational LCA in multi-stakeholder processes. Such efforts are critical to move from largely exploratory applications toward a coherent, standards-aligned methodological landscape for healthcare LCA.

## Figures and Tables

**Table 1:** Comparison of sector-specific LCA framework and practice in service category.

LCA-Phase	Subcategories	Care-Pathways	Service LCA Studies	Interpretation of Alignment
**Goal and Scope**	**Functional/declared unit**	Defined by care pathway characteristics (illness, severity, patient, location, duration).	Defined predominantly as *number of practices*, with strong preference for *one procedure*, occasionally *provision over time period (one year)*	Practice operationalises procedure-based rather than pathway-based
	**System boundary**	Flexible: cradle-to-gate or cradle-to-grave.	*Cadle-to-grave* and *door-to-door*.	Partial alignment; practice reflects both life cycle-based and spatial-based boundary interpretation
	**Life cycle stages**	Modular pathway structure (e.g., consultation, inpatient stay, surgery) incl. consumables, equipment, direct emissions, facilities data, staff travel, support services/admin. If applicable: pharmaceuticals, patient food.	Includes raw materials, production, transport, electricity use, pharmaceuticals, patient/staff transport, disposal.	Structural modelling mismatch between modular healthcare processes and patient flows rather than conventional upstream and downstream processes
**Life Cycle Inventory**	**Data collection**	Primary data for processes under control; secondary data otherwise.	Combination of hybrid and process-based data.	Broad methodological alignment
**Life Cycle Impact Assessment**	**Selection of impact categories and models**	Limited set: IPCC GWP 100, Global Water Footprint Standard, waste indicator.	Broad use: IPCC, TRACI, ReCiPe.	Guidance prescribes limited indicators, practice applies generic LCA methods
**Interpret-ation**	**Assessment**	Parameter, scenario, and model uncertainty; qualitative statements required, quantitative recommended.	Contribution, comparative, and sensitivity analyses frequently used.	Partial methodological convergence but no standardised reporting structure

**Table 2:** Comparison of sector-specific LCA framework and practice in category product.

LCA-Phase	Subcategories	GHG-P&M	Product LCA Studies	Interpretation of Alignment
**Goal and Scope**	**Functional/declared unit**	Flexible; defined per product or per use. Guiding questions on nature, quantity, duration, and context of use.	Typically *amount-based* or *use-based, followed by practice-based FU (predominantly one item/use/procedure)*. Pharmaceuticals also *Mass of Item*.	Practice shows greater heterogeneity and occasional practice-oriented framing
	**System boundary**	Cradle-to-grave, pharmaceuticals also cradle-to-gate.	Mostly cradle-to-grave; pharmaceuticals also cradle-to-gate	Broad alignment (cradle-to-grave approaches dominate both guidance and practice)
	**Life cycle stages**	Excludes: research and development, capital goods, overhead operations, corporate activities.	Staff transport, building energy use, and infrastructure were each included in only a single study.	Not all guidance-defined stages in practice included
**Life Cycle Inventory**	**Data collection**	Primary data for processes under control; secondary for external processes; focus on major contributors (>10%).	Predominantly process-based data; hybrid models occasionally used.	Broad alignment
**Life Cycle Impact Assessment**	**Selection of impact categories and models**	Limited to IPCC (carbon footprint).	Broader use (ReCiPe, IPCC, CML, others).	Standard limited to carbon, while studies apply multi-impact assessment
**Interpret-ation**	**Assessment**	Qualitative assessment of parameter, scenario, and model uncertainty; quantitative analysis encouraged; reporting required but no standardised format.	Sensitivity, comparative, and contribution analyses; heterogeneous reporting formats.	Similar methods but no harmonised reporting

## Data Availability

The extracted dataset of reviewed healthcare LCA studies and the detailed framework comparison are available in the Supplementary Materials. The study dataset is provided to ensure transparency and reproducibility of the analysis.

## Supplementary Materials

The following supporting information can be downloaded at: https://www.mdpi.com/article/10.3390/ijerph23070828/s1. The supplementary Excel file includes seven worksheets: S1—detailed overview and comparison of identified healthcare-specific LCA frameworks.; S2—extracted characteristics and methodological parameters of the reviewed healthcare LCA studies. S3—studies reported in the referenced meta-analyses used for completeness verification; S4—classification protocol and assignment rules used to categorise the reviewed studies according to product, service, and organisation categories; S5—hierarchical classification rules and decision criteria used to assign functional units to the developed functional unit clusters; S6—hierarchical classification rules and decision criteria used to assign system boundaries to the developed system boundary clusters; S7—supplementary visualisations related to Figure 5.

## Abbreviations

AHRQ	Agency for Healthcare Research and Quality
API	Active Pharmaceutical Ingredient
AWARE	Available Water Remaining
BMWE	German Federal Ministry for Economic Affairs and Energy (Bundesministerium für Wirtschaft und Energie)
CED	Cumulative Energy Demand
CF	Carbon Footprint
CML	Centrum voor Milieukunde Leiden Impact Assessment Method
CO_2_	Carbon Dioxide
EF method	Environmental Footprint Method (European Commission)
EPD	Environmental Product Declaration
EU	European Union
FU	Functional Unit
GHG	Greenhouse Gas
GHG-P&M	Greenhouse Gas Accounting Sector Guidance for Pharmaceutical Products and Medical Devices
GHG Protocol	Greenhouse Gas Protocol Corporate Accounting and Reporting Standard
ICHI	International Classification of Health Interventions
ILCD	International Reference Life Cycle Data System
IMPACT 2002+	Impact Assessment Method 2002+
IPCC	Intergovernmental Panel on Climate Change (Global Warming Potential Method)
ISO	International Organisation for Standardisation
LCA	Life Cycle Assessment
LCI	Life Cycle Inventory
LCIA	Life Cycle Impact Assessment
LCSA	Life Cycle Sustainability Assessment
OPS	Operationen- und Prozedurenschlüssel (German Procedure Classification)
PAS	Publicly Available Specification
PAS 2090	Pharmaceutical Products—Product Category Rules for Environmental Life Cycle Assessments
PCR	Product Category Rules
PCR-Blood	Product Category Rules for Blood and Blood-Derived Products
PCR-PPE	Product Category Rules for Disposable Surgical Drapes, Gowns, Air Suits and Face Masks
PCR-Pharma	Product Category Rules for Pharmaceutical Products and Processes
PEF	Product Environmental Footprint
PRISMA	Preferred Reporting Items for Systematic Reviews and Meta-Analyses
ReCiPe	Life Cycle Impact Assessment Method Combining Midpoint And Endpoint Indicators
Scope 1–3	Categories of Greenhouse Gas Emissions According to the GHG Protocol
TRACI	Tool for the Reduction and Assessment of Chemical and Other Environmental Impacts
USEtox	UNEP-SETAC Toxicity Model for Human Toxicity and Ecotoxicity
WHO	World Health Organisation
